# Propensity score matching in otolaryngologic literature: A systematic review and critical appraisal

**DOI:** 10.1371/journal.pone.0244423

**Published:** 2020-12-31

**Authors:** Aman Prasad, Max Shin, Ryan M. Carey, Kevin Chorath, Harman Parhar, Scott Appel, Alvaro Moreira, Karthik Rajasekaran

**Affiliations:** 1 Perelman School of Medicine, University of Pennsylvania, Philadelphia, PA, United States of America; 2 Department of Otorhinolaryngology, University of Pennsylvania, Philadelphia, PA, United States of America; 3 Biostatistics Analysis Center, University of Pennsylvania, Philadelphia, PA, United States of America; 4 Department of Pediatrics, University of Texas Health San Antonio, San Antonio, TX, United States of America; University of Mississippi Medical Center, UNITED STATES

## Abstract

**Background:**

Propensity score techniques can reduce confounding and bias in observational studies. Such analyses are able to measure and balance pre-determined covariates between treated and untreated groups, leading to results that can approximate those generated by randomized prospective studies when such trials are not feasible. The most commonly used propensity score -based analytic technique is propensity score matching (PSM). Although PSM popularity has continued to increase in medical literature, improper methodology or methodological reporting may lead to biased interpretation of treatment effects or limited scientific reproducibility and generalizability. In this study, we aim to characterize and assess the quality of PSM methodology reporting in high-impact otolaryngologic literature.

**Methods:**

PubMed and Embase based systematic review of the top 20 journals in otolaryngology, as measured by impact factor from the Journal Citations Reports from 2012 to 2018, for articles using PSM analysis throughout their publication history. Eligible articles were reviewed and assessed for quality and reporting of PSM methodology.

**Results:**

Our search yielded 101 studies, of which 92 were eligible for final analysis and review. The proportion of studies utilizing PSM increased significantly over time (p < 0.001). Nearly all studies (96.7%, n = 89) specified the covariates used to calculate propensity scores. Covariate balance was illustrated in 67.4% (n = 62) of studies, most frequently through p-values. A minority (17.4%, n = 16) of studies were found to be fully reproducible according to previously established criteria.

**Conclusions:**

While PSM analysis is becoming increasingly prevalent in otolaryngologic literature, the quality of PSM methodology reporting can be improved. We provide potential recommendations for authors regarding optimal reporting for analyses using PSM.

## Introduction

Randomized controlled trials (RCTs) provide the highest level of evidence when examining the effects of particular exposures or interventions on an outcome of interest. However, despite their ability to minimize selection bias and confounding, surgical RCTs are often viewed as expensive, challenging to conduct, limited in scope, and subject to extensive ethical considerations or debate [1–3]. These challenges may be particularly salient in subspecialties such as otolaryngology, as previous research has shown that RCTs comprise only 3.3–3.7% of published articles in the field [4, 5]. As a result, observational studies are more often utilized to retrospectively investigate clinical or epidemiologic data to make correlations regarding treatment efficacy, after which further confirmatory studies may be conducted. Unfortunately, observational studies are subject to treatment selection bias due to their lack of randomization and often become the basis of clinical practice despite the fact they do not provide the same level of scientific rigor as RCTs [6].

In an attempt to improve comparisons between cohorts in observational studies, statistical methodologies have been developed in order to reduce confounding when randomization is not possible [7]. The most commonly employed statistical technique to reduce bias is multivariable regression. However, while multivariable regression can help determine the effect size of an exposure on a given outcome and control for predetermined confounders, it is limited by model parsimony [8, 9]. In effect, multivariable regression is susceptible to ‘overfitting’ when too many potential confounders are included [8, 10].

Alternative approaches to reduce confounding may utilize *propensity scores* as a way to measure and balance baseline characteristics between two groups. A propensity score is defined as the probability (0 to 1) of receiving a treatment based on recorded baseline characteristics of an individual [11]. Several demographic or disease characteristic variables can be used at once to generate propensity scores for each individual in a study cohort. Unlike multivariable regression, propensity score models can be constructed with many more variables (potential confounders), which tend to improve the model’s inferential ability [12]. There are four primary methods to apply propensity score methodology. First, stratification by propensity score facilitates the comparison between exposure and outcome between smaller groups with more similar baseline characteristics [9]. Second, covariate adjustment can be used by including propensity scores as a dependent variable in a multivariable model [9]. Third, inverse probability of treatment weighting utilizes propensity scores to create differential weighting for each individual whereby the distribution of potential confounders is independent of exposure [9]. Fourth, and by far most common, propensity score matching (PSM) allows researchers to pair subjects in the control and treatment groups by matching individuals with similar propensity scores [11, 13, 14]. If done properly, analysis of outcome differences between treated and untreated participants following PSM can mimic that of an RCT [11, 15].

Because of its powerful implications, PSM is becoming increasingly common in many medical specialities, and presumably it will follow suit in otolaryngology [16]. Just as with any other research methodology, it is imperative that PSM methodology is comprehensively and accurately reported by authors. Failure to do so may result in biased results or limited statistical reproducibility and ultimately compromise patient care [17, 18]. Indeed, past research, both in non-surgical and surgical fields, has shown that the reporting and reproducibility of PSM methodology in medical literature may be at times flawed [14, 16, 19, 20]. Further, improper PSM reporting in high-impact surgical literature has been associated with increased odds of studies reporting statistically significant results [21].

Therefore, the primary objective of this study is to perform a systematic review of all published, high impact otolaryngologic literature using PSM. Accordingly, we aim to (1) analyze the quality of PSM methodological reporting, (2) assess the reproducibility of studies utilizing PSM, and (3) examine whether an association exists between improper PSM reporting and the odds of reporting significant results.

## Methods

This study was performed according to the guidelines set out in the Preferred Reporting Items for Systematic Review and Meta-Analysis (PRISMA) statement. As this study examined a de-identified collection of previously published manuscripts, a waiver of exemption was obtained from the Institutional Review Board of the University of Pennsylvania.

### Search strategy

We performed a systematic search through PubMed and Embase without limitation for language to identify otolaryngologic observational studies using PSM. Databases were queried for all studies containing “propensity” and “match*” ever published in the top 20 otolaryngologic journals, as ranked by the Journal Citations Reports in any year from 2012 to 2018 (Clarivate Analytics, 2018) [22]. The search was conducted on May 26, 2020 with no lower bound date restriction. The search was limited to human subjects.

One author performed the initial search. The titles and abstracts of all retrieved studies were independently reviewed by two independent authors, using Rayyan Systematic Review Software (Qatar Computing Research Institute, Doha). Studies were excluded if they met any of the following criteria: 1) were not an observational study; 2) were not surgical in nature; 3) did not use PSM analysis; 4) were case reports, letters to the editor, conference abstracts/posters, and articles without an abstract. Subsequently, each included manuscripts’ references were screened for additional articles.

### Data extraction and outcomes/measures

All studies included after initial abstract screening underwent blinded, full text review by two authors. Disagreements regarding exclusion criteria were resolved by a third-party with an advanced degree in epidemiology or biostatistics. Reporting quality was defined using guidelines adapted from Yao et al. and Grose et al. which have previously been used to evaluate PSM methodology in a variety of other fields [14, 16, 19, 21]. The following study characteristics were recorded: journal, journal impact factor, subspecialty, year, initial sample size, matched sample size, and collaboration with a statistician. Disagreements regarding data collection and coding were also resolved by a third party with advanced statistical knowledge. Collaboration with a statistician was defined as either having a co-author or an individual mentioned in the acknowledgments section with (1) a confirmed degree in biostatistics or epidemiology, such as an MPH, MS, or PhD; or (2) an affiliation with a department of biostatistics or similar quantitative statistics area, which is consistent with prior study methodology [23]. Specific methodological characteristics relevant to PSM were collected from each article and their definitions with examples of proper reporting are listed in **Table 1**. Additionally, we recorded whether studies found statistically significant results for their primary outcome. If the primary outcome was unclear, the outcome given the most attention in the discussion section by the length of text it was examined was selected [21]. PSM reproducibility was assessed based on inclusion of four reporting criteria as established by Lonjon et al and others [14, 24–27]: (1) the algorithm used for matching; (2) the matching ratio; (3) whether replacement was used in the matching process (4) whether the statistical tests used to compare PSM groups assumed independent or paired groups. Only those studies satisfying all four criteria were deemed fully reproducible.

**Table 1:** Definitions and examples of key methodological components of propensity score matching.

Methodological component	Definition	Example from Included Studies
Covariates reported with justification	These covariates represent the variables which are included in the PSM model. Omitting a true confounder (if available) may bias results. Justification for these covariates provides rationale to readers regarding each selection and allows readers to independently assess if important variables were omitted from the propensity score model.	“Eighty-four pairs of patients were successfully matched using 14 covariates: sex, age, affected side, body mass index (BMI), concomitant symptoms such as vertigo and tinnitus, lifestyle factors such as drinking and smoking, systemic disease such as hypertension and diabetes, audiometric curves, the average of pure tone audiometry evaluations pre and post treatment, and time to treatment initiation” [28]
		“We selected covariates known to affect treatment selection. These primarily included sociodemographic characteristics (age, highest education level, and marital status). These variables have demonstrated associations with the ability to travel large distances to specific medical centers, or to affect how severe a patient’s disease was at presentation Additionally, we included variables believed to be related to the outcome but not necessarily the treatment to reduce bias…[etc]” [29]
Summary Statistics	Summary statistics including baseline numbers and percentages for the overall study sample and the post-matched sample.	Table 3 [30]
Covariate Balance	Covariate balance is used in order to assess whether the two matched groups (treatment and control) differ substantially based on the covariates described above. If a large difference remains, this indicates the two groups have not been ideally matched and that confounding is still present.	“To test the covariate balance after propensity-score matching, we calculated standardized differences to compare the baseline characteristics of patients between the cetuximab-based RT and CCRT groups for both unmatched and propensity score–matched groups. A standardized difference of >10% was defined as out of balance.” [31]
Estimation of Propensity Score	Specifications regarding the type of regression model used to generate propensity scores.	“…[P]ropensity scores were estimated for each patient using a multivariable logistic regression adjusting for all covariates.^20^” [32]
Sensitivity Analysis	Sensitivity analyses can be used to assess for residual confounding, particularly due to bias that was unaccounted for during matching. Specifically, it determines the extent to which an omitted covariate could impact the treatment effect.	“Formal sensitivity analysis was performed as described elsewhere.” [33]
Matching Algorithm	The method through which patients in each group are matched based on their calculated propensity scores (e.g. Greedy algorithm vs Optimal algorithm).	“The second step was matching patients 1:1 via the nearest-neighbor matching strategy without replacement, with 0.2 SD of the logit of the propensity score as the caliper value.” [34]
Matching Ratio	This describes the ratio of untreated subjects that are matched to treated subjects (e.g. 1:1, 2:1, 3:1, etc.). 1:1 is the most commonly used ratio.	
Caliper Specifications	The caliper is the maximum distance or value that propensity scores between matched subjects is allowed differ.	
Replacement	This describes whether a single untreated subject is allowed to be matched with more than 1 treated subject (matching with replacement) or to only 1 treated subject (matching without replacement)	
Paired Statistical Methods	The statistical method used to assess treatment effects. Statistical tests can either assume samples are independent or paired.	“We used Kaplan-Meier methods and multivariable Cox proportional hazards regression models to evaluate OS…The Cox models were then stratified by matched pair, and CIs were calculated using robust SEs to account for correlated observations.” [35]

### Statistical methods

We calculated the yearly proportion of studies using PSM by obtaining the following ratio: # of studies using PSM / total # of publications in the sampled journals. The Cochran–Armitage test for trend was used to assess the presence of a statistically significant linear trend in proportion of studies using PSM over time. Continuous variables were assessed for normality using the Shapiro-Wilk test and summarized with median and quartiles. Univariate logistic regression, Chi-square analyses, Fisher’s Exact Tests and the Wilcoxon Rank Sum Tests were used to assess the association between bibliometric factors and study reproducibility and the association between PSM methodological components and statistically significant findings. The latter was also assessed using multivariate logistic regression. Predictor selection for the multivariable analyses was determined by a univariate p value <0.20. For this analysis, a mixed model was selected to account for both fixed effects and random effects in the explanatory variables. In particular, we set the journal variable to a random intercept to help account for within-journal and between-journal variability. In all relevant instances, a two-sided type 1 error rate of 0.05 was used to indicate statistical significance. All calculations were performed using STATA 14.2 (STATA Corp, College Station). All data used for statistical analysis can be found via an online data repository through Harvard Dataverse [36].

## Results

### Overall study characteristics

**Fig 1:**
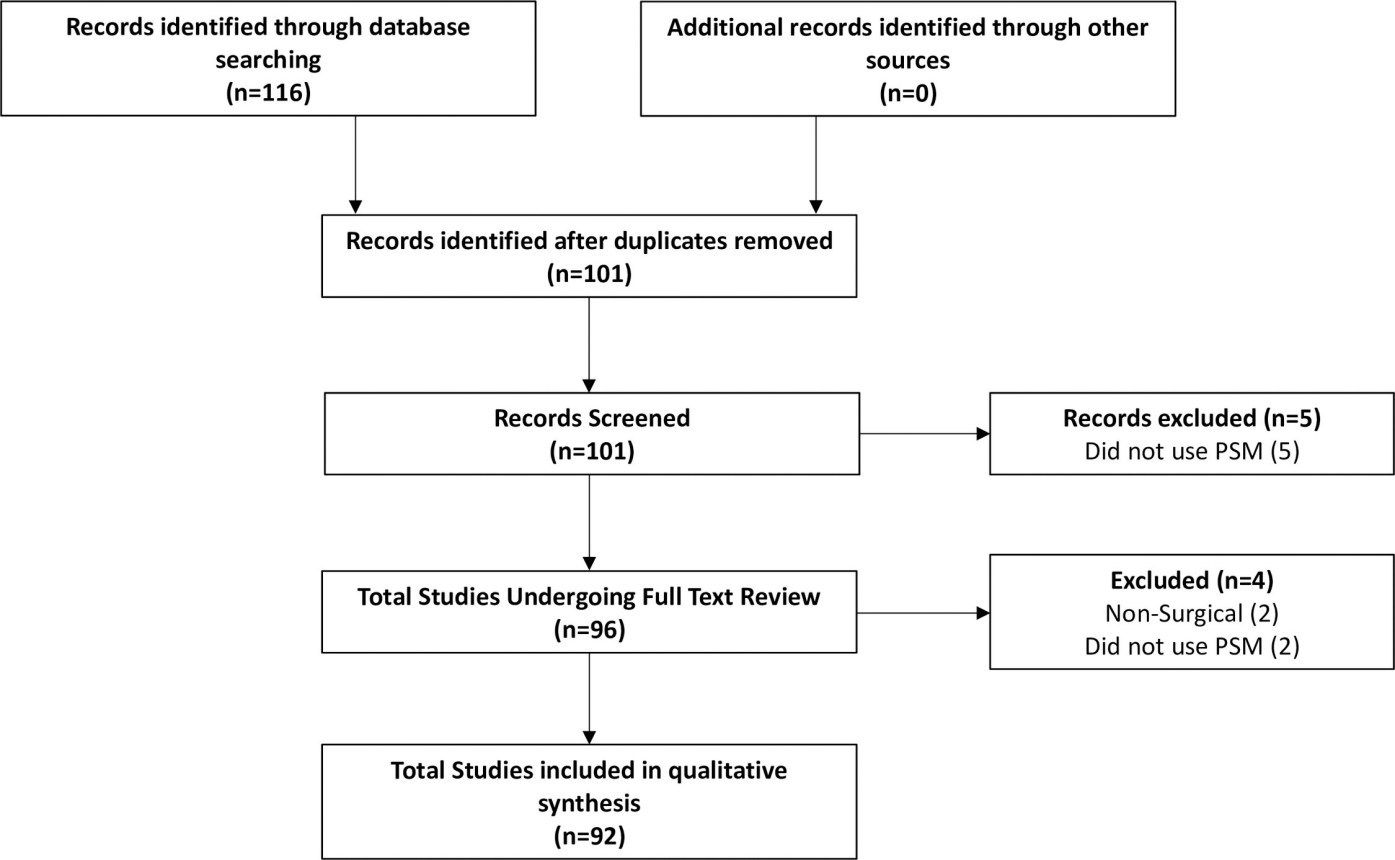
PRISMA flow diagram of our search strategy demonstrating the total number of studies reviewed and reasons for exclusion.

**Fig 2:**
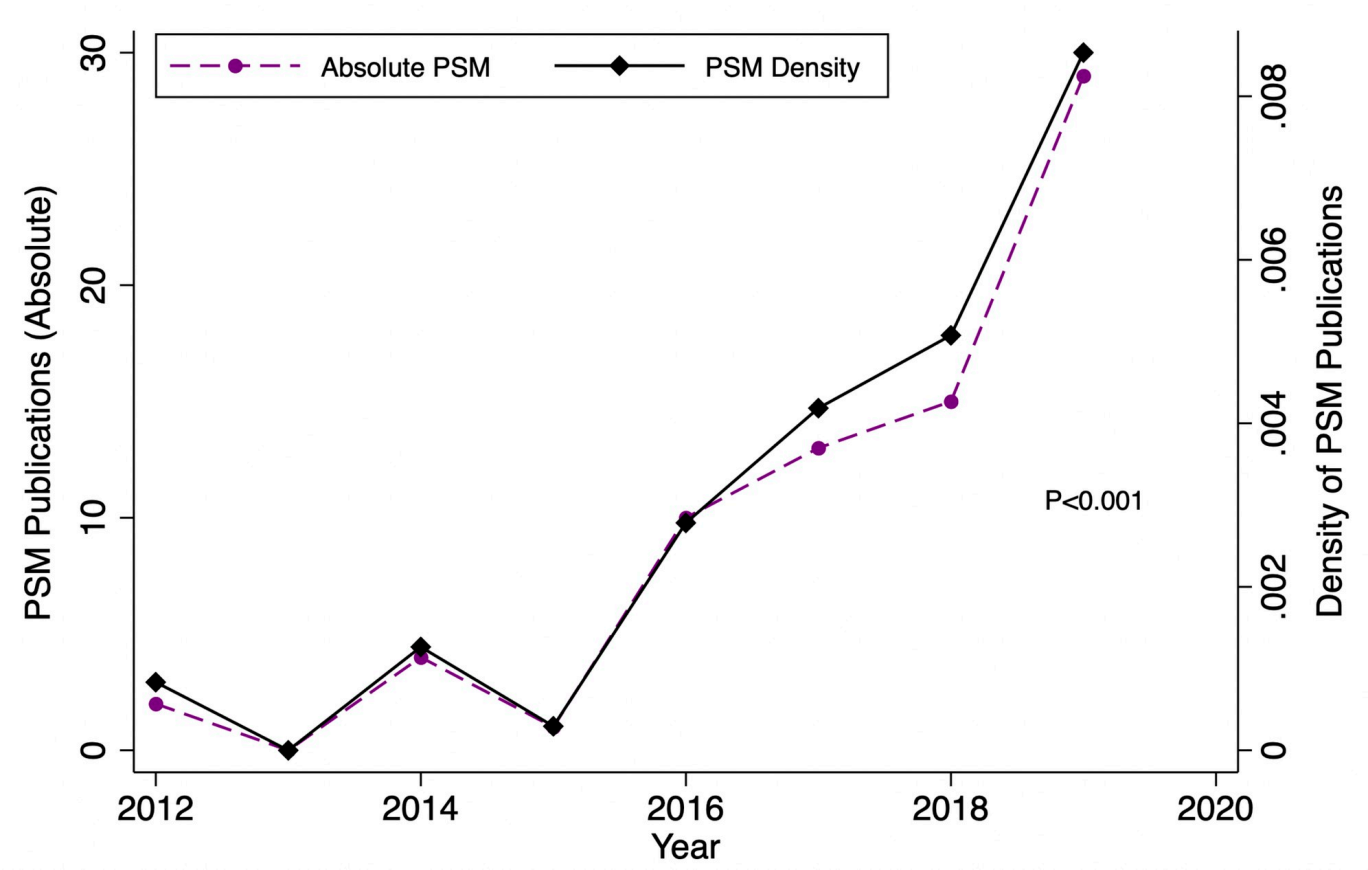
Trend analysis depicting the absolute and relative increase in the number of studies using PSM methodology over time.

The initial search rendered 101 articles (**Fig 1**). After duplicate removal and application of exclusion criterion during abstract review, five articles were excluded. Subsequently, a total of 96 articles underwent full text review. With respect to abstract review, the authors were in substantial agreement (κ = 0.71) regarding article inclusion. Upon review, an additional four articles not satisfying inclusion criteria were excluded because they were non-surgical in nature [37, 38] or used propensity score methods aside from PSM [39, 40], ultimately yielding 92 articles eligible for final analysis. **Table 2** outlines the general characteristics of all 92 studies. The impact factors of the included journals ranged from 1.29 to 3.50 (median: 2.38). Eighty-three (90.2%) of articles included in the analysis were author -described retrospective cohort studies. PSM analysis was most common in head in neck surgery articles (50.0%, n = 46) as compared to other subspecialties within otolaryngology. Fourty-four (47.8%) studies had a statistician as a co-author. Furthermore, study publication years ranged from 2012 to 2020, and we found that the proportion of studies employing PSM increased significantly over time (p<0.001) (**Fig 2**).

**Table 2:** Characteristics of included studies.

Study Characteristic	*N = 92*
**Year of Publication**	
2012 (%)	2 (2.2)
2013 (%)	0 (0.0)
2014 (%)	4 (4.4)
2015 (%)	1 (1.1)
2016 (%)	10 (10.9)
2017 (%)	13 (14.1)
2018 (%)	15 (16.3)
2019 (%)	29 (31.5)
2020 (%)	18 (19.6)
**Journal with Impact Factor (2018 Impact Factor)** ^*^	
*Acta Otolayngologica*, *1*.*286 (%)*	3 (3.3)
*American Journal of Rhinology & Allergy*, *2*.*015 (%)*	1 (1.1)
*Clinical Otolaryngology*, *2*.*377 (%)*	3 (3.3)
*European Archives of Oto-Rhino-Laryngology*, *1*.*750 (%)*	8 (8.7)
*Head & Neck*, *2*.*442 (%)*	23 (25.0)
*International Forum of Allergy & Rhinology*, *2*.*521 (%)*	4 (4.4)
*JAMA Otolaryngology–Head & Neck Surgery*, *3*.*502 (%)*	17 (18.5)
*Otolaryngology–Head and Neck Surgery*, *2*.*310 (%)*	12 (13.0)
*The Laryngoscope*, *2*.*343 (%)*	21 (22.8)
**Study Design**	
Retrospective Cohort (%)	83 (90.2)
Prospective Cohort (%)	2 (2.2)
Case Control (%)	7 (7.6)
**Subspecialty**	
General (%)	7 (7.6)
Head & Neck Surgery (%)	46 (50.0)
Laryngology (%)	2 (2.2)
Otology (%)	16 (17.4)
Rhinology (%)	11 (12.0)
Thyroid (%)	10 (10.9)
**Presence of Statistician Co-Author**	
Yes (%)	44 (47.8)
No (%)	36 (45.0)
Unknown (%)	12 (12.4)
**Median Initial Cohort Size (IQR)**	1438 (403–6900)
**Matched Cohort Size (IQR)**	924 (218–3948)
**Percent of Cohort Matched (%)**	50.6 (33.3–64.4)

^*^ The following journals did not return articles that fit our inclusion criteria

Rhinology, The Journal of Vestibular Research: Equilibrium & Orientation, Dysphagia, Ear and Hearing, Hearing Research, Journal of Vestibular Research, Trends in Hearing, JARO, Journal of Otolaryngology-Head & Neck Surgery, Otology & Neurotology, Audiology and Neurotology, International Journal of Audiology, European Archives of Oto-Rhino-Laryngology, Otolaryngologic Clinics of North America, Current Opinion in Otolaryngology & Head and Neck Surgery, Journal of the American Academy of Audiology, Journal of Voice, International Journal of Pediatric Otorhinolaryngology, and Trends in Amplification.

### Quality of PSM methodological reporting

Full details regarding the reporting of the methodological components of included studies are outlined in **Table 3**. Nearly all (96.7%, n = 89) studies reported the covariates used in constructing the propensity score model; 37.1% (n = 33) of such studies provided justification for their selections. Fifty-nine (64.1%) studies did not provide summary statistics for their pre- and post-matched populations. Most studies (67.4%, n = 62) reported on the technique used to illustrate covariate balance, most frequently through p-values (42.4%). Standardized differences were reportedly used in 22.8% of studies. Lastly, 4 studies (4.4%) reported the use of a sensitivity analysis.

**Table 3:** Characteristics and quality of PSM reporting (n = 92).

Methodology Characteristic	*(N = 92)*
**Covariates Reported**	
Yes (%)	89 (96.7)
Justification Given (%)	33 (37.1)
No Justification Given (%)	56 (62.9)
No (%)	3 (3.3)
**Summary Statistics Provided for Pre / Post-Matched Populations (%)**	33 (35.9)
**Balance Technique**	
Standardized Differences (%)	21 (22.8)
<10% (%)	15 (71.4)
<20% (%)	1 (4.7)
< 25% (%)	1 (4.7)
Not Reported (%)	4 (19.0)
P Values (%)	39 (42.4)
Graphically (%)	2 (2.2)
Not Reported (%)	30 (32.6)
**Regression Model Provided** (%)	57 (62.0)
**Sensitivity Analysis Provided** (%)	4 (4.4)
**Matched Sample Size Included** (%)	75 (81.5)
Type of Matching Algorithm Used *	
Greedy NN no caliper (%)	14 (15.2)
Greedy NN within caliper (%)	36 (39.1)
Reported how it was generated (%)	20 (55.5)
Failed to Report how it was generated (%)	16 (44.4)
Optimal matching (%)	1 (1.1)
Reported how it was generated (%)	1 (100.0)
Failed to Report how it was generated (%)	0 (0.0)
Digit (%)	1 (1.1)
Not Reported (%)	40 (43.5)
**Use of Statistical Methods that account for Matched Data?** *	
Yes (%)	14 (15.2)
No (%)	63 (68.5)
Not Reported (%)	15 (16.3)
**Ratio Used** *	
1:1 (%)	67 (72.8)
1:2 (%)	6 (6.5)
1:3 (%)	4 (4.3)
Other (%)	11 (12.0)
Not Reported (%)	4 (4.3)
With or Without Replacement? *	
Without (%)	21 (22.8)
With (%)	1 (1.1)
Not Reported (%)	70 (76.0)
**Reproducibility Score**	
0/4 (%)	1 (1.1)
1/4 (%)	11 (12.0)
2/4 (%)	29 (31.5)
3/4 (%)	35 (38.0)
4/4 (%)	16 (17.4)

*Criteria used to determine Reproducibility Score

### Reproducibility of studies using PSM

Sixteen (17.4%) studies were found to be fully reproducible, with the remaining (82.6%) studies lacking at least 1 of the 4 elements necessary for complete reproducibility. The Greedy Nearest Neighbor method within a specified caliper was the most common matching algorithm used (39.1%); of studies using that algorithm, 44.4% did not, however, report how the caliper was generated. The most commonly used matching ratio was 1:1 (72.8%). Seventy-seven (83.7%) studies clearly reported the statistical tests and methodology they used to compare matched pairs. Of those, 14 (15.2%) used methods that accounted for the matched nature of the data. Twenty-two (23.9%) studies reported whether or not they used replacement in their propensity score model (**Table 3**). Ten (66.7%) studies that were fully reproducible included a statistician co-author, as opposed to 52.3% (n = 34) of studies that were not fully reproducible.

Following univariate logistic regression, journal impact factor was significantly associated with PSM reproducibility. We found that there was 2.76 times higher odds of studies being fully reproducible for each unit increase in its associated journal impact factor (CI: [1.06–7.22]) (**Table 4**).

**Table 4:** Associations between bibliometric factors and PSM reproducibility.

Methodologic Characteristic	Proportion of Studies (n, %)^‡^			Overall P-value†	Univariate Logistic Regression		
	Overall	Fully Reproducible	Not Fully Reproducible		OR	95% CI	P Value
	(N = 92)	(N = 16)	(N = 76)				
Inclusion of Statistician Co-Author*	44 (55.0)	10 (66.7)	34 (52.3)	0.314	1.83	0.56–5.93	0.318
Study Design				0.272			
Journal				0.616			
Impact Factor	2.38 (2.31–2.44)	2.44 (2.33–3.5)	2.34 (2.31–2.44)	0.163	2.76	1.06–7.22	**0.039**
Subspecialty				0.344			
General	7 (7.6)	0 (0.0)	7 (9.2)				
Head & Neck	46 (50.0)	7 (43.8)	39 (51.3)				
Laryngology	2 (2.2)	0 (0.0)	2 (2.6)				
Otology	16 (17.4)	3 (18.8)	13 (17.1)				
Rhinology	11 (12.0)	2 (12.5)	9 (11.8)				
Thyroid	10 (10.9)	4 (25.0)	6 (7.9)				
Year of Publication				0.398	0.92	0.68–1.24	0.581
Matched Cohort Size	924 (218–3948)	2179 (282–5174)	924 (188–3494)	0.455	1.00	1.00–1.00	0.921
Original Cohort Size	1438 (403–6900)	3560 (507–10265)	1291 (403–4796)	0.301	1.00	1.00–1.00	0.992

*The presence of a statistician co-author could not be identified in 11 studies, and those studies were excluded from analyses.

†*Overall p* values were calculated by chi-squared analysis, Fisher’s Exact Test, or the Wilcoxon Rank Sum Test where appropriate

^‡^ Reported as # studies reporting methodological characteristic/total # of studies in subgroup (%) for categorical variables and median (first quartile, third quartile) for continuous non-parametric variables.

### Associations between PSM methodology reporting and finding significant results

Employing univariate logistic regression to assess associations between study PSM reporting characteristics and findings of significant results, we found studies that were fully reproducible were 78% less likely to report significant results compared to studies that were not fully reproducible (OR: 0.22, CI: [0.07–0.69]). Additionally, studies that reported the regression model used to create the PSM model were 72% less likely to find significant results (OR: 0.28, CI: [0.10–0.85]). Following mixed effect multivariable logistic regression analysis, there were no statistically significant associations between covariates and the presence of statistically significant results (**Table 5**).

**Table 5:** Associations between PSM methodology reporting and statistical significance of primary outcome.

Methodologic Characteristic	Proportion of Studies (n, %)^‡^			Overall P-value†	Univariate Logistic Regression			Multivariate Logistic Regression*		
	Overall	Reported Significant Results	Did not Report Significant Results		OR	95% CI	P-Value	OR	95% CI	P-Value
	(N = 92)	(N = 66)	(N = 26)							
Algorithm Reported	52 (56.2)	34 (51.5)	18 (69.2)	0.123	0.47	0.18–1.24	0.127			
Ratio Reported	87 (94.6)	63 (95.5)	24 (92.3)	0.549	1.75	0.28–11.1	0.550			
Replacement Reported	22 (23.9)	12 (18.2)	10 (38.5)	**0.040**	0.36	0.13–0.97	**0.044**			
Paired Stats Method	77 (83.7)	56 (84.9)	21 (80.8)	0.633	1.33	0.41–4.36	0.634			
Reproducibility Score				**0.006**						
0 or 1	12 (13.0)	7 (10.6)	5 (19.2)		Ref.	-	-			
2 or 3	64 (69.6)	52 (78.8)	12 (46.2)		3.10	0.84–11.45	0.090			
4	16 (17.4)	7 (10.6)	9 (34.6)		0.56	0.12–2.52	0.447			
Fully Reproducible	16 (17.4)	7 (10.6)	9 (34.6)	**0.006**	0.22	0.07–0.69	**0.009**	0.26	0.07–1.02	0.053
Covariates Included	89 (96.7)	63 (95.5)	26 (100)	0.269						
Covariates Justified	33 (35.9)	20 (30.3)	13 (50.0)	0.076	0.43	0.17–1.10	0.079	0.87	0.28–2.68	0.810
Illustrated Co-Variate Balance	62 (67.4)	43 (65.2)	19 (73.1)	0.465	0.69	0.25–1.88	0.467			
Use of SMD	18 (19.6)	11 (16.7)	7 (26.9)	0.264	0.54	0.18–1.60	0.268			
Regression Model Reported	57 (62.0)	36 (54.6)	21 (80.8)	**0.020**	0.28	0.10–0.85	**0.024**	0.32	0.10–1.00	0.051
Goodness of Fit Test	3 (3.3)	2 (3.0)	1 (3.9)	0.840	0.78	0.07–9.00	0.843			
Sensitivity Analysis	4 (4.4)	3 (4.6)	1 (3.9)	0.882	1.19	0.12–12.0	0.882			
Baseline Characteristics	33 (35.9)	20 (30.3)	13 (50.0)	0.076	0.44	0.17–1.10	0.080	0.43	0.14–1.33	0.142

OR = Odds Ratio; CI = Confidence Interval

†Overall *p* values were calculated by chi-squared analysis, Fisher’s Exact Test, or the Wilcoxon Rank Sum Test where appropriate

^‡^ Reported as # studies reporting methodological characteristic/total # of studies in subgroup (%) for categorical variables and median (first quartile, third quartile) for continuous non-parametric variables.

*For the multivariate analysis, a mixed effect logistic model with random effect for journal was used

## Discussion

PSM techniques help researchers reduce bias due to confounding by generating propensity scores based on preselected baseline characteristics, and subsequently matching individuals with similar scores. By accounting for covariates that predict whether individuals receive a treatment, investigators are able to minimize treatment assignment bias and more accurately measure treatment effects in observational studies. This is a powerful tool that can allow researchers to more appropriately consider future clinical trials. We present the first systematic review of the otolaryngology literature to evaluate the use and reporting of PSM methodology. We found systematic underreporting of PSM methodological components in the otolaryngological literature, which is in accordance with findings of other studies in different fields [14, 21, 41–43].

While the use of PSM has significantly increased over the study period, many studies did not adequately report their methodology which would have helped to ensure unbiased interpretation of results, high levels of external validity, and feasible reproducibility. Consistent with findings from similar studies in other fields, the majority of studies did not report justification for the inclusion of chosen covariates in creating propensity score models [16, 21, 42]. This is recommended in order to facilitate transparency and unbiased interpretation of treatment effects. Similarly, we found that only 4.5% of studies reported the use of a sensitivity analysis, which can inform researchers of the robustness of their findings to omitted covariates or biases [44]. Indeed, one of the limitations of PSM is that it only controls for covariates included in the propensity score. Unlike true randomization, residual confounding can still bias results when important covariates are not identified *a priori*, or not available for use in the propensity score [43, 45]. Additionally, following matching, it is often standard practice to assess any residual inter-group differences through reporting covariate balance. In our study, the most common way such balance was illustrated was through p-values calculated from significance testing, as opposed to using standardized mean differences (SMD). Using p-values to assess covariate balance may be biased due to the differences in sample sizes between the matched and pre-matched samples, whereas SMD are independent of sample size [11]. Furthermore, we found that the majority of studies did not provide summary statistics for their pre-matched and post-matched samples, similar to findings shown in other fields which showed that as low as 0% of studies did so [14, 19, 21]. Such information may be important for interpretation of the generalizability of results, especially when baseline characteristics of the matched population differs significantly from those of the original [16].

The importance of study reproducibility has been emphasized in recent years, because lack of reproducibility in PSM may hinder subsequent investigation, either confirmatory or meta-analytic [46]. To assess reproducibility, we examined 4 factors previously established in the literature, as outlined by Lonjon et al. and others [14, 20, 24–26]. We found that 17.4% of studies met all 4 of these criteria, whereas Lonjon et al. found that 10% of studies were fully reproducible in surgical literature as a whole [14]. Specifically, a number of different matching algorithms and matching ratios for PSM exist, each of which may produce different results; thus, in order to best interpret published results, it is important to clarify which of these are used. Furthermore, knowledge of the replacement technique used in the analysis may be relevant for readers, as matching with replacement introduces variance that should subsequently be accounted for by the use of specific statistical methods [47]. Lastly, while our results indicate that most studies were clear about which statistical tests were used, we also found that most did not use tests that account for matched data. Because treatment and control patients of a matched sample are not independent [27], it is recommended that statistical methods comparing treatment effects account for matching. Past literature has shown that not doing so may introduce bias by resulting in inappropriate Type 1 error rates [48]. Appropriate statistical tests that account for matched data include the paired *t* test, Wilcoxon signed rank test, McNemar test, stratified log-rank test, or Cox proportional hazards models stratified on matched pairs [27, 48, 49].

With this in mind, we initially hypothesized that statistician involvement in a study would increase the likelihood of reproducibility due to the complexity of PSM methodology. Interestingly, however, we did not find inclusion of a statistician co-author to be predictive of study reproducibility, as indicated by the results of our univariable and multivariable logistic regression. On the other hand, journal impact factor was found to be significantly associated with reproducibility. It is possible that journals with higher impact factors have more rigorous review processes or publication guidelines, thereby increasing the likelihood that article methodology is sufficiently detailed. However, this is likely only true to a certain extent, as research has shown that although impact factor may be a reasonable indicator of journal quality as rated by physicians, such correlations are not perfect. Thus, other unmeasured or bibliometric variables may contribute to our findings [50].

We also sought to understand the effects of PSM reporting on likelihood of articles publishing significant results of their primary outcome, as perhaps studies with less rigorous reporting were more likely to report significance due to lax methodology. Using univariate logistic regression, we found there to be a significant association between studies that were not fully reproducible and reporting of significant results. While this association was not found to be significant (p = 0.053) in our mixed-effects multivariable logistic regression, albeit by slim margins, it nevertheless may indicate that reproducibility may represent a proxy for appropriate experimental methodology. In other words, studies neglecting to report core components of reproducibility may have been less fastidious, be it intentionally or not, in ensuring the robustness of other components of their methodology. This is especially pertinent in study designs using propensity score matching, given the numerous steps where errors can occur, which can be further compounded by PSM’s iterative nature.

The results of the data presented herein demonstrate that the reporting of PSM methodology in otolaryngology has room for improvement. This is especially important given that the prevalence of PSM utilization is increasing by year as shown in **Fig 2** and **Table 2**. We propose several recommendations for authors to consider. First, covariates used to generate propensity scores and rationale for these selections should be specified. Second, in order to ensure matched pairs do not differ substantially, balance between covariates may be assessed through use of standardized mean differences, thereby minimizing biased treatment effects. Third, authors may also consider conducting a sensitivity analysis to further ensure robust matching. Fourth, all four criteria for reproducibility of a propensity-matched analysis should be reported, as discussed above. Fifth, in order to assess the external validity of a study, readers may find it useful if the matched sample size is specified and baseline characteristics or summary statistics for pre- and post-matched populations are provided [14]. A summary of examples of adequate reporting are presented in **Table 1**. Additionally, although no significant difference in reproducibility was found between studies with or without statistician coauthors, it is advisable for individuals with strong statistical knowledge and background to be involved in the study and be listed as a co-author if appropriate, as implementation of PSM can be a complex process. However, as a reminder to readers, PSM is still limited by its ability to only control for confounders that are known and measurable. Well-designed RCTs, by contrast, are able to control for all confounders by virtue of treatment randomization and thus remain the gold standard in clinical research.

The results of this study should be interpreted in the context of its study design and in consideration of several important limitations. For one, we were unable to identify the presence of a statistician co-author in eleven studies, and our analysis of this variable may therefore be incomplete. In addition, our multivariable logistic analysis of the effect of PSM methodology reporting on statistical significance was based solely on the primary outcome as stated by the authors. In certain situations, studies with non-significant primary outcomes may have had statistically significant secondary outcomes which may be of clinical importance which would have been overlooked by our model. We also acknowledge that some of the included studies may have hypothesized equivalence between two groups or treatments, in which case lack of statistical significance may have been a positive result which would have been misclassified in our analysis. Furthermore, we acknowledge that this investigation is likely subject to a degree of publication bias in that studies with positive results compose the majority of published literature. This must be considered a potential confounder in our analysis of statistically significant results [51]. Lastly, this study was limited to higher impact otolaryngologic journals and may therefore not be completely reflective of this specialty’s literature as a whole. Still, this study serves as the first reported systematic review of the use of PSM in high impact otolaryngology journals and identifies several areas for improvement.

## Conclusion

PSM represents a valuable tool for researchers to minimize bias and confounding in analyses of observational studies. In this systematic review of high-impact otolaryngology journals, the prevelance of PSM analysis was found to be increasing by year. Despite this, the quality of PSM methodological reporting in otolaryngology can be improved in order to ensure unbiased interpretation of results. We provide authors with recommendations to maximize scientific rigor in such studies. Authors, reviewers, and readers alike should be cognizant of such considerations when designing or interpreting studies utilizing PSM methodology.

## Supporting information

S1 Table PRISMA checklist. Completed PRISMA checklist for our literature review.
(DOC)
