# Correction: Propensity score matching in otolaryngologic literature: A systematic review and critical appraisal

**DOI:** 10.1371/journal.pone.0250949

**Published:** 2021-04-27

**Authors:** Aman Prasad, Max Shin, Ryan M. Carey, Kevin Chorath, Harman Parhar, Scott Appel, Alvaro Moreira, Karthik Rajasekaran

There is an error in affiliation for author Karthik Rajasekaran. The correct affiliation is as follows:

Karthik Rajasekaran ^2,5^

**2** Department of Otorhinolaryngology, University of Pennsylvania, Philadelphia, PA, United States of America, **5** Leonard Davis Institute of Health Economics, University of Pennsylvania, Philadelphia, PA, United States of America.
